# Supplementary material for: Single-cell RNA-seq of rheumatoid arthritis synovial tissue using low-cost microfluidic instrumentation
Source: Nat Commun. 2018 Feb 23;9:791. doi: 10.1038/s41467-017-02659-x (PMC5824814; doi:10.1038/s41467-017-02659-x)
Supplement: Supplementary file 1 — Supplementary Information [file 41467_2017_2659_MOESM1_ESM.pdf]

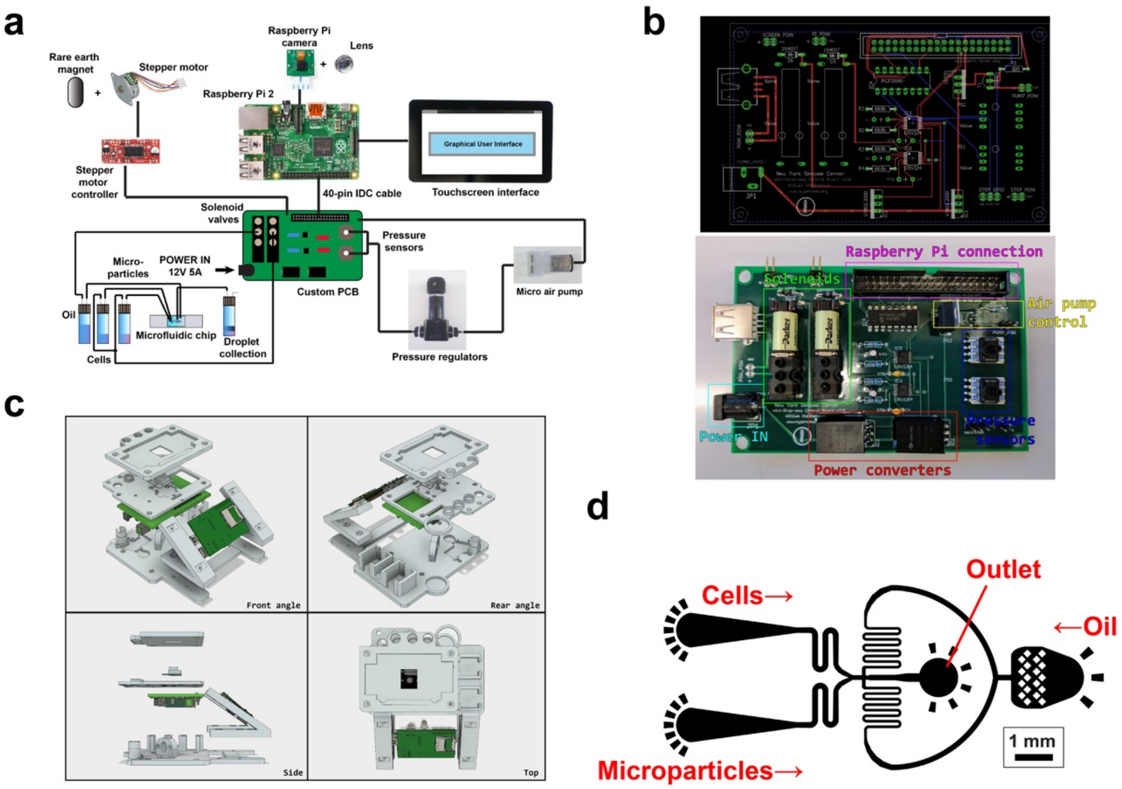

Supplementary Figure 1 | Microfluidic control instrument design and microfluidic chip design. a) Component diagram of the instrument. The instrument is controlled with a Raspberry pi 2 model B single board computer that interfaces with the components through a custom designed printed circuit board (PCB). **b)** Circuit layout (top) and image (bottom) of the completed PCB. **c)** Multi-angle view of the 3D printed instrument frame. **d)** Microfluidic chip design. Cell and microparticle inlets have equal hydrodynamic resistance up to the junction with the bifurcated oil channel.

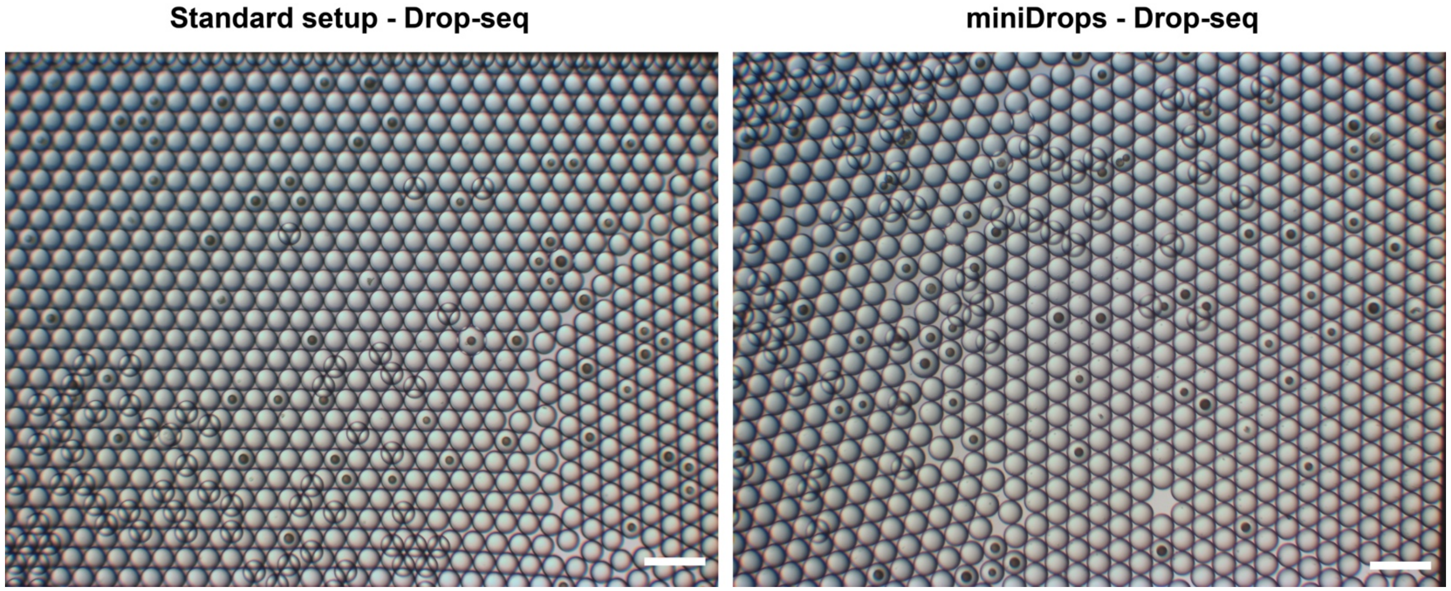

Supplementary Figure 2 | Droplet comparison. 4X Microscope images of droplets produced from standard Drop-seq setup and miniDrops microfluidic control instrument. Scale bar in both images is 250 µm.

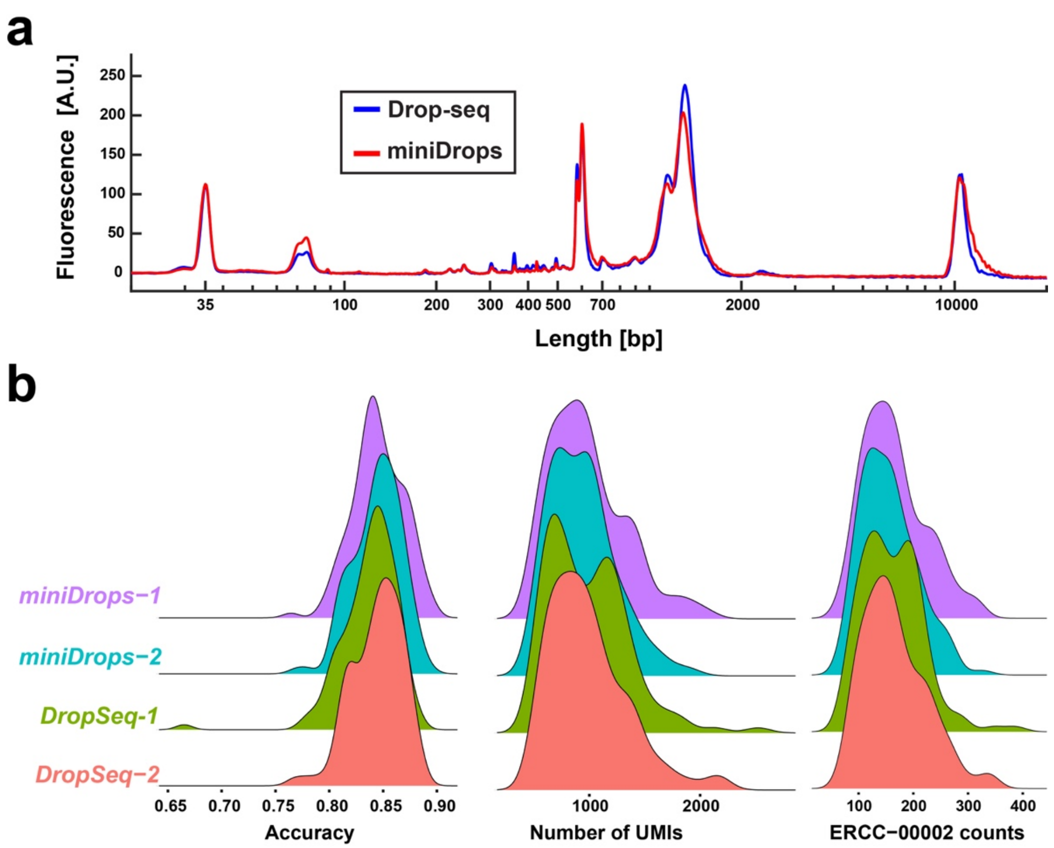

Supplementary Figure 3 | Benchmark experiments. a) Bioanalyzer traces of cDNA generated after reverse transcription from ERCC spike-in controls for the standard Drop-seq setup and the miniDrops instrument. b) Accuracy, number of detected UMIs and counts of ERCC-00002 transcript across both instrument setups in replicate.

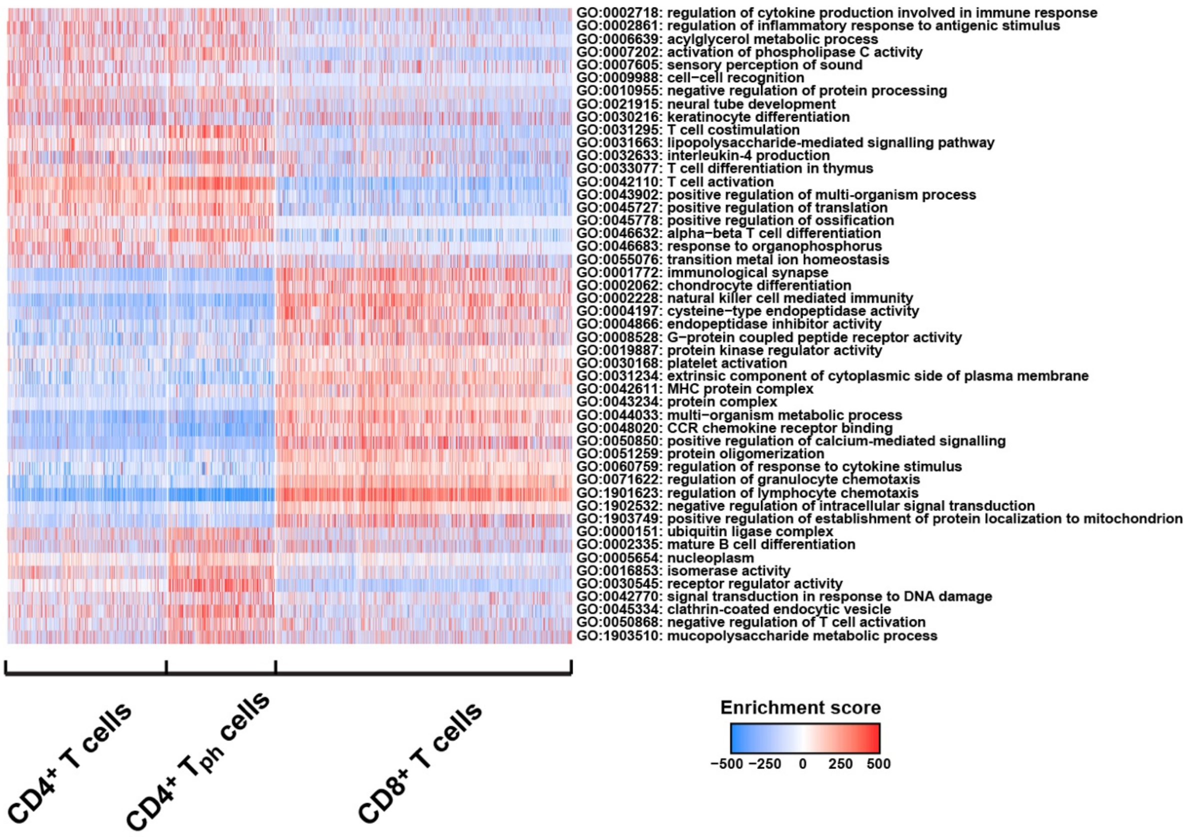

Supplementary Figure 4 | T cell GO enrichment. Pathway and gene set overdispersion analysis on the three T cell populations identified via unbiased clustering of the single cell RNA-seq data. The enrichment score corresponds to each cells' first principle component loading from pathway analysis as computed in *pagoda*.

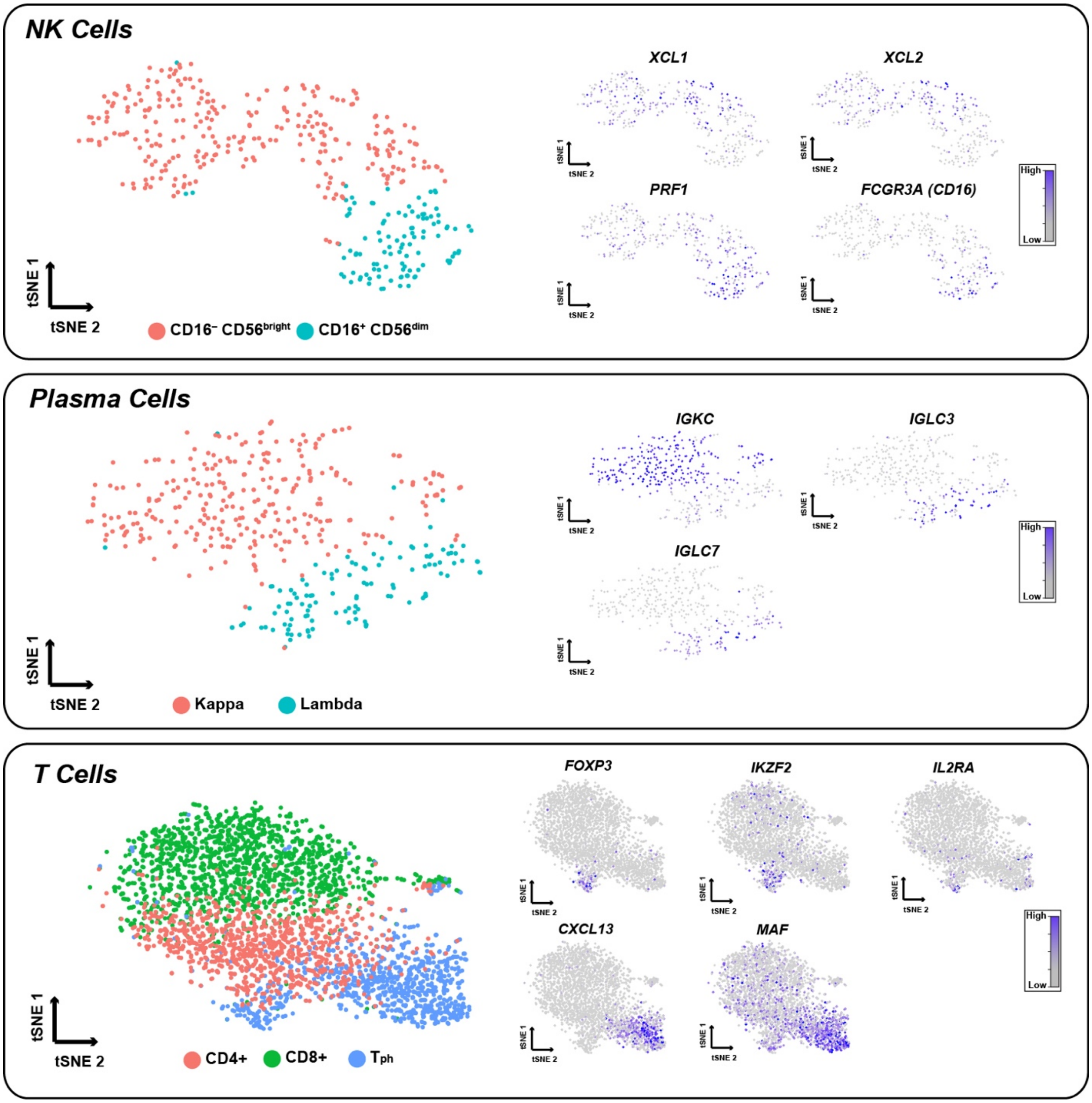

Supplementary Figure 5 | Further characterization of NK, Plasma, and T cells. Independent analyses of NK, plasma, and T cell populations revealed further heterogeneity within each broad cell type. The NK cells separated into CD16$^-$/CD56^{bright} and CD16$^+$/CD56^{dim} subsets characterized by expression of XCL1/2 and PRF1/FCGR3A respectively. The plasma cells separated into two groups defined by antibody light chain usage (IgA kappa+ vs IgA lambda+). Within the T cell class, we also observe an additional rare population of T cells that were initially grouped with T$_{PH}$, defined by upregulation of FOXP3, IL2RA, and IKZF2 that likely represents a distinct population of CD4+CD25+ regulatory T cells.

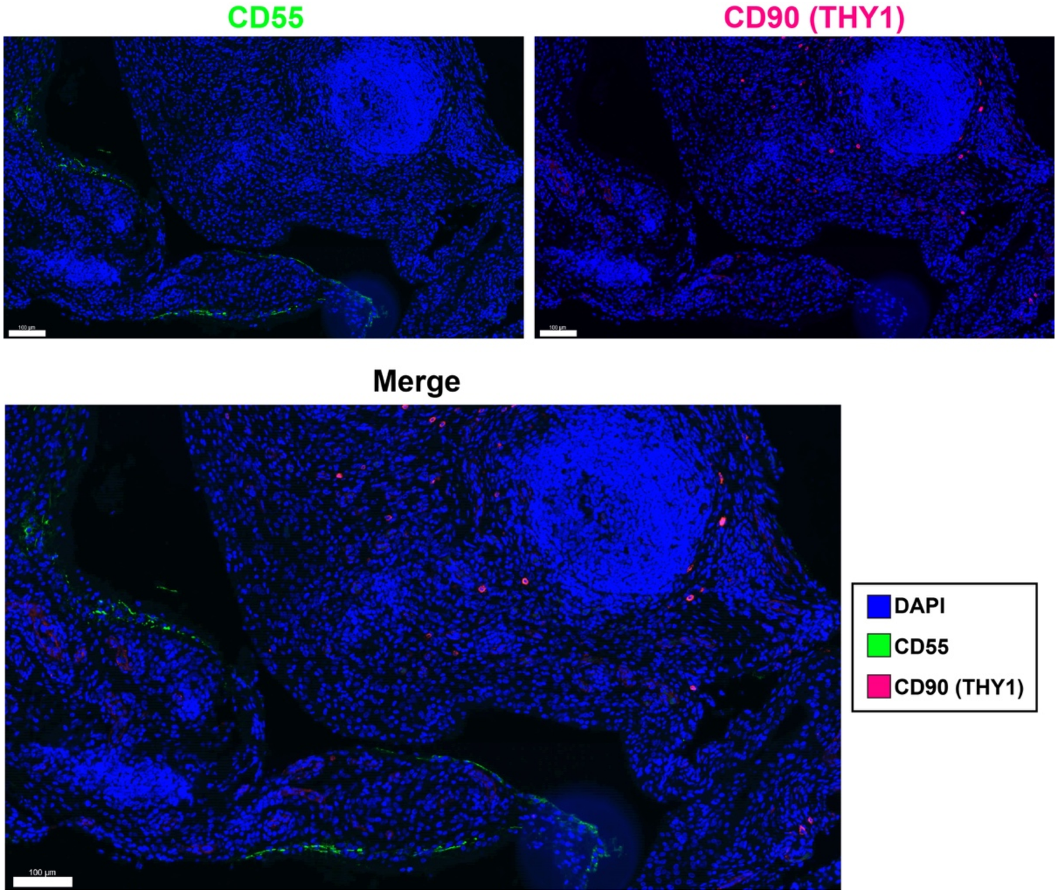

Supplementary Figure 6 | Dual stain immunofluorescence of RA synovial tissue. CD55 and CD90 dual stain of synovial tissue. Merge image shows the presence of CD55 and CD90 stained cells within the same tissue section. Scale bars are 100µm for each image.

Supplementary Table 1 | Bill of materials (BOM) for the microfluidic control instrument (miniDrops).

Part No.	Item Description	SKU	Cost/Unit [$]	Quantity	Cost [$]	Supplier
1	Raspberry Pi 2 model B	95Y1948	35.00	1	35.00	Newark Element14
2	Micro SD Card 8GD SD10	SDSDQUAN-008G-G4A	9.89	1	9.89	Amazon.com
3	Raspberry Pi Touchscreen	49Y1712	60.00	1	60.00	Newark Element14
4	7mm collimating laser diode lens	B00PPSJJ40	1.54	1	1.54	Amazon.com
5	Raspberry Pi camera	77Y6521	18.50	1	18.50	Newark Element14
6	mX7 Solenoid valve	961-712331-000	42.00	2	84.00	Parker Hannafin Precision Fluidi
7	Airtrol Regulator	V800-30 W/K	36.30	2	72.60	Hi-Tech Pneumatics
8	Micro air pump	AP-2P01	65.00	1	65.00	Smart products
9	4-phase 5VDC unipolar stepper motor	237825	9.95	1	9.95	Jameco
10	EasyDriver stepper motor driver	ROB-12779	14.95	1	14.95	SparkFun
11	Custom PCB	N/A	25.00	1	25.00	Royal Circuit Solutions
12	Honeywell TruStability pressure sensor	SSCDLNN015PGAA5	32.87	2	65.74	DigiKey
13	DC/DC converter	V7805-2000R	10.68	2	21.36	DigiKey
14	Solenoid driver DRV104	296-15746-1-ND	5.62	2	11.24	DigiKey
15	LD1117V50-DG transistor (IC3 on PCB)	497-12821-5-ND	0.57	1	0.57	DigiKey
16	68Kohm RES	PPC68.1KZTR-ND	0.52	4	2.08	DigiKey
17	1uF CAP	493-12567-3-ND	0.11	2	0.22	DigiKey
18	470pF CAP	1286PH-ND	0.25	2	0.50	DigiKey
19	22ohm RES (R5 on PCB)	CF14JT22R0CT-ND	0.10	1	0.10	DigiKey
20	2.1mm Barrel Jack Connector	PRT-00119	1.25	1	1.25	SparkFun
21	MCP3008	856	3.75	1	3.75	Adafruit
22	2N4401 transistor	2N4401D75ZCT-ND	0.15	1	0.15	DigiKey
23	40-PIN IDC connector	2222	1.00	1	1.00	Adafruit
24	1N4007 DIODE	1N4007FSCT-ND	0.09	2	0.18	DigiKey
25	Disc1 magnet N42	D403	0.29	4	1.16	K&J Magnetics
26	Disc2 magnet N42	D46	0.79	1	0.79	K&J Magnetics
27	60W AC-to-DC switching power supply 12V 5A	1952370	18.95	1	18.95	Jameco
28	PLA 3D printing plastic (@ $41/750g or $41/95m)	Ultimaker PLA Black	0.43	21.13	9.12	fbrc8
29	Cylinder magnet	D48-N52	1.28	1	1.28	K&J Magnetics
29.5	USB Female Type A SMD Connector	PRT-09011	1.25	1	1.25	SparkFun
30	Machine screw 8-32, 1/4"	2094346	0.069	8	0.552	Jameco
31	Machine screw 8-32, 1/2"	106797	0.079	4	0.316	Jameco
32	Machine screw 4-40, 1/2"	106810	0.079	8	0.632	Jameco
33	Machine screw 2-56 x 1/4"	38173	0.099	4	0.396	Jameco
34	Nut 8-32	51553	0.059	4	0.236	Jameco
35	Nut 4-40	40943	0.059	4	0.236	Jameco
36	Nut 2-56	38165	0.059	4	0.236	Jameco
37	Internal tooth washer #4	106850	0.059	2	0.118	Jameco
38	Flat washer #4	106826	0.065	2	0.13	Jameco
39	Hex standoff, male-female, 8-32, 1/2", aluminum	93505A452	0.56	4	2.24	McMaster-Carr
40	Hex standoff, male-female, 8-32, 3/4", aluminum	93505A454	0.61	8	4.88	McMaster-Carr
41	Round Standoff, female-female, 8-32, 20.6mm or 0.8125"	93330A457	0.54	4	2.16	McMaster-Carr
42	Spacer round, 4.75mm or 0.187" tall	92510A421	0.27	2	0.54	McMaster-Carr
43	Thread to barb, 1/8-27NPT, 7/16" Hex classic Barb	1810-6005	0.418	2	0.836	Nordson Medical/Value plastics
44	Tee tube fitting with 200 series barbs, 3/32" (2.4 mm) ID	T220-2	0.246	4	0.984	Nordson Medical/Value plastics
45	Elbow tube fitting with 200 series barbs, 3/32" (2.4 mm) ID	L220-2	0.246	21	5.166	Nordson Medical/Value plastics
46	3 Foot USB Data Sync and Power Charge Cable	2135064	2.49	2	4.98	Jameco
47	Tygon tubing E-3603, 3/32" ID, 5/32" OD	EW-06407-73	67	0.06	4.02	Cole-Parmer
48	USB flex light	B00D2ZDY2Q	9.99	1	9.99	Amazon.com
49	26G steel tubing 45 deg angle, 1" long	NE-236-304-1-45	-	5	-	New England Small Tube
50	Buna N O-ring, 8mm x 2mm	1RJD1	-	5	-	McMaster-Carr
51	1.8 mL Cryo vials, internal threading	363401PK	-	-	-	Thermofisher Scientific
52	4.5 mL Cryo vials, internal threading	363452PK	-	-	-	Thermofisher Scientific

TOTAL: ***$575.77***
