# Supplementary material for: Single-cell RNA-seq of rheumatoid arthritis synovial tissue using low-cost microfluidic instrumentation
Source: Nat Commun. 2018 Feb 23;9:791. doi: 10.1038/s41467-017-02659-x (PMC5824814; doi:10.1038/s41467-017-02659-x)
Supplement: Supplementary file 3 — Description of Additional Supplementary Files [file 41467_2017_2659_MOESM3_ESM.pdf]

Description of Additional Supplementary Files

File Name: Supplementary Movie 1
Description: Video capture from the microfluidic control instrument. Cell and microparticle encapsulation into nanoliter volume droplets. Microparticles can be seen entering the junction from the bottom left. Cells can be seen entering the junction from the top left. At the junction, aqueous droplets are formed through fluorinated oil co-flow, and individual droplets can be visualized just after the junction. All flow is from left to right.

File Name: Supplementary Data 1
Description: Single cell RNA-seq dataset. Genes that are differentially expressed in each of 13 identified clusters as compared to all other clusters by the non-parametric Wilcoxon rank sum test implemented in Seurat v2.1.

File Name: Supplementary Data 2
Description: Bulk RNA-seq dataset. Genes that are differentially expressed in the bulk RNA-seq data between CD55+ and CD90+ fibroblast populations as determined by DESeq2.
